# Nitrogen dynamics and phytoplankton community structure: the role of organic nutrients

**DOI:** 10.1007/s10533-017-0351-8

**Published:** 2017-06-15

**Authors:** Grigorios Moschonas, Richard J. Gowen, Ruth F. Paterson, Elaine Mitchell, Brian M. Stewart, Sharon McNeill, Patricia M. Glibert, Keith Davidson

**Affiliations:** 1 Scottish Association for Marine Science, Argyll, Oban, PA37 1QA Scotland, UK; 2 Fisheries and Aquatic Ecosystems Branch, Agriculture Food and Environmental Science Division, Agri-Food and Biosciences Institute, Newforge Lane, Belfast, BT9 5PX UK; 3 Horn Point Laboratory, University of Maryland Center for Environmental Science, PO Box 775, Cambridge, MD 21613 USA

**Keywords:** Dissolved organic nitrogen, Phytoplankton, Nitrogen uptake, Community composition

## Abstract

Dissolved organic nitrogen (DON) is recognised as an important N source for phytoplankton. However, its relative importance for phytoplankton nutrition and community composition has not been studied comprehensively. This study, conducted in a typical Scottish fjord, representative of near-pristine coastal environments, evaluates the utilisation of DON and dissolved inorganic nitrogen (DIN) by different microbial size fractions and the relationship of phytoplankton community composition with DON and other parameters. The study demonstrated that DON was important in supporting phytoplankton throughout the yearly production cycle. The higher-than-expected urea uptake rates and large fraction of the spring bloom production supported by DON suggested that organic N not only contributes to regenerated production and to the nutrition of the small phytoplankton fraction, but can also contribute substantially to new production of the larger phytoplankton in coastal waters. Multivariate statistical techniques revealed two phytoplankton assemblages with peaks in abundance at different times of the year: a spring group dominated by *Skeletonema* spp., *Thalassiosira* spp., and *Pseudo*-*nitzschia* spp. group *delicatissima*; and a summer/autumn group dominated by *Chaetoceros* spp., *Scrippsiella* spp., and *Pseudo*-*nitzschia* spp. group *seriata*. The multivariate pattern in community composition and abundance of these taxa was significantly correlated with the multivariate pattern of DON, urea, dissolved free amino acids, DIN, temperature, salinity, and daylength, with daylength and urea being particularly important, suggesting both physical and chemical controls on community composition.

## Introduction

It is important to understand the processes that influence phytoplankton community composition and abundance, in the context of both natural phytoplankton species succession (Smayda 1980) and undesirable disturbance (Tett et al. 2007). Nitrogen (N), as nitrate (NO_3_^−^), nitrite (NO_2_^−^), and ammonium (NH_4_^+^), has historically been considered the main limiting nutrient for phytoplankton growth and biomass in coastal waters (Howarth 1998; Howarth and Marino 2006; Ryther and Dunstan 1971; Vitousek and Howarth 1991). As such, it is potentially a principal determinant of phytoplankton community composition and abundance: changes in the concentration (Gowen et al. 2012a), ratio to other nutrients, and proportion of different chemical forms (Davidson et al. 2012) of N can lead to changes in phytoplankton community composition and abundance (Gowen et al. 2012a; Glibert et al. 2016 and references therein), potentially promoting the growth of harmful species (Glibert et al. 2005) or altering the carbon export flux of coastal seas (Bronk et al. 2007).

Dissolved organic N (DON) is also increasingly recognized as an N source for phytoplankton. Up to ~70% of DON in the marine environment may be bioavailable (Berman and Bronk 2003; Bronk 2002; Sipler and Bronk 2015) in labile (e.g. urea, amino acids) and semi-labile forms (Glibert et al. 2006), but it was previously considered a substrate that predominantly supported bacterial production (Bronk et al. 2007; Zehr and Ward 2002). However, DON, especially urea, which is used in nitrogenous fertilisers and can contribute to anthropogenic nutrient enrichment, has been linked to the growth of phytoplankton including harmful algal blooms (HABs) (Anderson et al. 2002; Glibert et al. 2005, 2006, 2014a). Dissolved free amino acids (DFAA), as well as peptides, have also been demonstrated to be important for phytoplankton nutrition (see Mulholland and Lomas 2008; Mulholland and Lee 2009).

Although it is increasingly clear that DON serves as an N source for both bacteria and phytoplankton (Antia et al. 1991; Bronk et al. 2007; Lønborg et al. 2009b; Pete et al. 2010), there is little information on the relative utilisation of different forms of inorganic and organic N over an annual cycle or possible links to phytoplankton community composition (Glibert et al. 1982a, 2016 and references therein, Davidson et al. 2014, 2012).

The utilisation of different forms of DON by different phytoplankton species and size fractions and its effect on phytoplankton community composition has not been studied comprehensively, especially using rigorous statistical techniques to support observations. Now that we know that autotrophs utilise DON in competition with heterotrophs, and that DON concentrations globally are significant, with a mean of 4.4 ± 0.5 µmol N L^−1^ (Letscher et al. 2013), a major research question is which organisms are utilising the different forms of organic N, when, and in what form (Sipler and Bronk 2015). Different features of DON biogeochemistry have been studies around the British Isles (e.g. Davidson et al. 2013; Fitzsimons et al. 2011; Moschonas et al. 2015; Tappin et al. 2010). The areas around the British Isles where DON has been studied are the North Sea, English Channel Irish Sea and adjacent shelf as well as English estuaries and Scottish fjordic sea-lochs (for examples of studies in each area see Moschonas et al. (2015)). Few of the studies, however, have directly measured N uptake by the microbial community.

The objectives of this study were therefore to evaluate the importance of DON for phytoplankton nutrition relative to that of inorganic N in two different microbial community size fractions (< and >10 µm) and to identify patterns in phytoplankton community composition and abundance associated with chemical and physical factors using rigorous statistical analysis. We chose to conduct this study in Loch Creran, Scotland, because it represents a near-pristine coastal location, providing a useful baseline for the study of anthropogenic pressures on coastal ecosystems; and many aspects of its hydrography, biology, and chemistry have been previously studied (Booth 1987; Fehling et al. 2006; Lønborg et al. 2009b; Tett et al. 2011) allowing the new knowledge generated in this study to be placed in a wider context.

## Methods

### Sampling

**Fig. 1:**
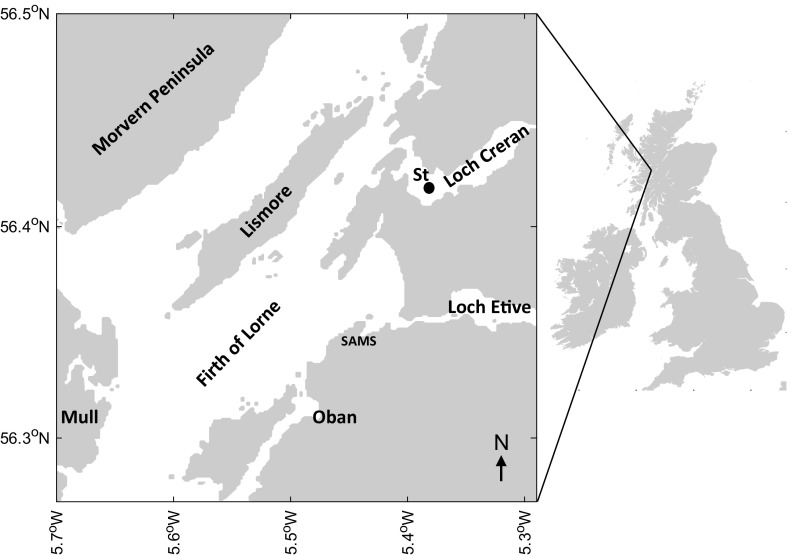
Map of Loch Creran with sampling station (St) and adjacent geography

Sampling was carried out between 18 January and 12 December 2012 from the Scottish Association for Marine Science (SAMS) research vessel *Seol Mara*, at a sampling station (56°31′N, 5°22.6′W) in Loch Creran (Fig. 1). Sampling was conducted in daylight before noon on 20 different dates at roughly 2 week intervals, sufficient to resolve the major events in the annual cycle of plankton such as the spring bloom. Vertical profiles of temperature, conductivity, and photosynthetically active radiation were recorded at each station using a SBE 19 CTD (Sea-Bird Electronics). Practical salinity (dimensionless) and in situ temperature were converted to absolute salinity (g kg^−1^), conservative temperature (°C), and potential density (kg m^−3^) per the International Thermodynamic Equation of Seawater 2010 in the Gibbs SeaWater Oceanographic Toolbox (IOC et al. 2010; McDougall and Barker 2011). Accordingly, the salinity variable in this study is absolute salinity with units of g kg^−1^, rather than practical salinity which is dimensionless (McDougall and Barker 2011). Temperature and salinity values are presented hereafter as average potential temperature and average absolute salinity for the upper 25 m of the water column. Water was collected using a 5 L Niskin-type bottle from the depth of the fluorescence maximum (if present), or 10 m. Water was immediately pre-filtered (200 μm mesh) to exclude larger zooplankton, and was stored for ~1 to 1.5 h in opaque thermally insulated polypropylene containers until return to the laboratory.

### Chemical analyses

The pre-filtered water was used for the determination of urea, DFAA, and dissolved inorganic nutrient concentrations. On return to the laboratory the water sample was filtered through pre-combusted 10 or 25 mm glass fibre filters (PALL A/E, 0.7–1 μm nominal pore size prior to combustion) using clean plastic syringes, and stored frozen in polyethylene acid-washed sample bottles at −20 °C until analysis. Dissolved inorganic nutrients were analysed on a five Channel QuAAtro autoanalyser (Seal Analytical), configured for the simultaneous determination of dissolved inorganic N (DIN), silicon (DIS) and phosphorus (DIP) in sea water (Seal Analytical, 2011a, b, c, d, e).

Urea analysis was performed using the manual diacetyl monoxime method (Mulvenna and Savidge 1992), adapted for room temperature (Goeyens et al. 1998), use of a single reagent, and low sample volumes (Revilla et al. 2005). The working reagent was made up of 1 part of solution A (diacetylmonoxime, thiosemicarbazide) and 3.2 parts of solution B (sulphuric acid, ferric chloride) as described in Revilla et al. (2005). Samples were allowed to thaw overnight, then 4 mL of each replicate were transferred to 15 mL polypropylene centrifuge tubes, the reagent added and the solutions mixed by vortex before being incubated at 22 °C in the dark for 72–84 h (Goeyens et al. 1998). Subsequently, absorbance was measured by spectrophotometry at 520 nm on an Evolution 300 UV–Vis Spectrophotometer (Thermo Scientific).

Total DFAA concentrations were determined by the *o*-phthalaldehyde fluorometric method (Parsons et al. 1984), optimised for the use of 3-mercaptopropionic acid in the reagent, as this gives better reliability than mercaptoethanol (Aminot and Kérouel 2006). The working reagent was made up of 20 mL of solution A (*o*-phthalaldehyde, ethanol), 400 mL of solution B (pH adjusted ortho-boric acid solution) and 1 mL 3-mercaptopropionic acid as described in the above studies. Samples were allowed to thaw overnight, and 5 mL of each replicate transferred to 15 mL polypropylene centrifuge tubes. Following reagent addition, each solution was vortex mixed and allowed to stand for 2 min before its fluorescence was measured on a Trilogy laboratory fluorometer (Turner Designs). Calibration was with glycine standards and the DFAA concentrations were expressed as a glycine equivalent (Parsons et al. 1984; Tada et al. 1998).

Determination of dissolved organic carbon (DOC) and DON concentrations followed on-board filtration through pre-combusted 25 mm glass fibre filters (0.7–1 μm nominal pore size prior to combustion), using acid-washed glass syringes, acidified with 50 μL 85% orthophosphoric acid. Samples were stored in sealed pre-combusted glass containers at 4 °C in the dark until analysis (Davidson et al. 2007). This was performed using high temperature catalytic oxidation with non-dispersive infrared and chemiluminescence detections on a Shimadzu TOC-Vcph total organic carbon (TOC) module connected to a Shimadzu TNM-1 total N (TN) module. When samples are filtered, TDN is measured by the TN module. For each run the measurements were quality checked with a deep seawater reference (Florida Strait at 700 m, batch 9-2009, 41–44 μmol L^−1^ DOC, 32.25–33.75 μmol L^−1^ TN). Concentrations of DOC were determined directly after the acidified samples were sparged with air to remove dissolved inorganic carbon. Concentrations of DON were calculated indirectly by subtracting DIN from TDN, and the propagated error was calculated by combining errors in quadrature (Taylor 1997).

For the determination of chlorophyll (Chl) concentrations, one batch of samples was filtered directly through pre-combusted 47 mm glass fibre filters (PALL A/E, 0.7–1 μm nominal pore size prior combustion) under gentle vacuum (<100 to 150 mmHg pressure differential), while a second batch was first filtered through pre-washed polycarbonate membranes (10 μm pore size), to retain the >10 μm size fraction with only the filtrate then being secondary filtered through pre-combusted 47 mm glass fibre filters (PALL A/E, 0.7–1 μm nominal pore size) in an identical manner to above. All samples were stored frozen at −20 °C in 15 mL centrifuge tubes until analysis. Prior to analysis, pigments were extracted in 90% neutralised acetone overnight, then Chl concentration was determined on a Trilogy fluorometer (Turner Designs) using the Chl acidification technique and corrected for phaeopigments (Arar and Collins 1997).

### Phytoplankton enumeration

Water samples for phytoplankton identification and enumeration were fixed with Lugol’s iodine solution to 1% final concentration, and stored refrigerated in opaque amber plastic bottles. Prior to identification, 50 mL aliquots from each sample were settled in Hydro Bios chambers for 20–24 h, then examined under a Carl Zeiss Axiovert inverted microscope at ×20 magnification. Authorities for genera and species were taken from WoRMS (2016). Phytoplankton cells were identified to genus, or species level where possible, and abundance values were expressed as number of cells L^−1^. Morphotype groups of the genera *Pseudo*-*nitzschia* Peragallo (Fehling et al. 2006) and *Chaetoceros* Ehrenberg (Hasle and Syvertsen 1997) were also identified.

### Size fractionated N uptake of nitrate, ammonium, urea and DFAA

Uptake rates of N were determined using the ^15^N tracer technique (Dugdale and Wilkerson 1986; Glibert and Capone 1993). After a 1.5 h transit to the laboratory, water was transferred into transparent 250 mL polycarbonate bottles. Sets of four bottles received one of ^15^N labelled (98+%) NH_4_Cl, NaNO_3_, urea, and an algal amino acid mixture (Sigma-Aldrich). We aimed to achieve a tracer concentration of 10% of ambient N concentration (Dugdale and Goering 1967), estimated from historical N concentrations at the sampling station; as a result, the tracer concentrations rarely exceeded 20% of ambient. Immediately after the tracer additions, bottles were incubated in tanks with flow-through seawater drawn continuously from the sea to simulate in situ temperature and covered with appropriate light filters to simulate in situ light intensity. Following a 2–2.25 h incubation, half of the replicates for each ^15^N labelled compound were filtered at <100–150 mmHg pressure differential directly through pre-combusted 25 mm glass fibre filters (PALL A/E; 0.7–1 μm nominal pore size before combustion), while the other half were first filtered through pre-washed polycarbonate membranes (10 μm pore size), and then through pre-combusted 25 mm glass fibre filters to retain the <10 μm size fraction. Goldman and Dennett (1985) found that avoiding air exposure of filters minimises intracellular material loss from cell stress. Therefore, for each sample, when most of the water had filtered through, and before the filter was exposed to air (as in Goldman and Dennett 1985), it was rinsed with ‘aged’ filtered seawater to wash away any residual dissolved nutrients (Glibert and Capone 1993; Lipschultz 2008). Filters were stored frozen at −20 °C in the dark until analysis.

Prior to analysis, the samples were thawed at room temperature, dried overnight at 60 °C, and then folded into tin foil capsules (Sercon). Particulate organic carbon (POC), particulate organic N (PON), and the N stable isotope composition of each sample were measured on a PDZ Europa 20-20 Stable Isotope Analyser connected to an ANCA-NT system, calibrated with l-Isoleucine standards (Sigma-Aldrich) and modified for analysis of low mass samples (Owens and Rees 1989). Absolute and specific N uptake rates were calculated per Dugdale and Wilkerson (1986). The calculation was not corrected for isotope dilution (Glibert et al. 1982b; Kanda et al. 1987) by N regeneration, which could result in an underestimate of the NH_4_^+^ and urea uptake rates (Glibert et al. 1982b; Hansell and Goering 1989).

### Statistical analysis

Multivariate patterns of phytoplankton community composition and abundance and their relationship to environmental variables were analysed by applying Multidimensional Scaling (MDS), Hierarchical Agglomerative Clustering (Cluster), and Spearman rank correlation (BIO-ENV) coupled to permutation (RELATE) analysis on Bray-Curtis similarity matrices of sample or taxon standardised data on the software package PRIMER (Clarke and Ainsworth 1993; Clarke 1993; Clarke and Green 1988).

To statistically select groups of phytoplankton with similar patterns in seasonal distribution, namely taxa that increased with time, cluster analysis was applied to a between-taxon Bray–Curtis similarity matrix. This matrix was generated from taxon-standardised abundance of a subset of the ‘most important’ taxa, that is, the taxa that contributed the most to the abundance of each sample. This subset was limited to 15 taxa, which removed the ‘rarer’ taxa and enabled the effective between-taxa analysis. The removal of the rarer taxa is necessary for the statistical exploration of species similarities (Clarke and Ainsworth 1993; Clarke 1993; Clarke and Green 1988).

Following the selection of taxa belonging to different groups, their abundances were log(x + 1) transformed to balance the influence of low and high abundances of taxa on multivariate patterns. Then, MDS analysis was applied to the between-sample Bray–Curtis similarity matrix generated from the transformed abundances to visualise the multivariate pattern in community composition and abundance. The similarity matrix generated from the selected subset of taxa was compared to a similarity matrix of all enumerated taxa. This revealed that the multivariate pattern of community composition and abundance based on the subset of taxa was representative of that based on all enumerated taxa. It was then analysed for statistical correlation with a number of environmental variables by applying BIO-ENV.

## Results

From all measured environmental variables that could influence phytoplankton community composition (Table 1), seven (Table 2) significantly correlated (p < 0.02) with the multivariate pattern of community composition and abundance. From the 10 best correlations, the variables that emerged as dominant were urea, daylength, temperature, DFAA, DON, salinity, and DIN (Table 2).

**Table 1:** Environmental variables that could influence phytoplankton community composition and abundance and were included in the multivariate analyses

Variable	Units
DIN	μmol N L^−1^
DON	μmol N L^−1^
Urea	μmol N L^−1^
DFAA	μmol N L^−1^
DOC	μmol L^−1^
DIP	μmol L^−1^
DIS	μmol L^−1^
Temperature	°C
Salinity	g kg^−1^
Light attenuation	m^−1^
Density difference 1–25 m	kg m^−3^
Tidal range	m
Daylength	h

**Table 2:** Results of BEST analysis (p < 0.02)

Spearman rank correlation	DIN (μmol N L^−1^)	DON (μmol N L^−1^)	Urea (μmol N L^−1^)	DFAA (μmol N L^−1^)	Temperature (°C)	Salinity (g kg^−1^)	Daylength (h)
0.580			X		X		X
0.548		X	X		X		X
0.547			X	X	X		X
0.542		X	X	X	X		X
0.541			X				X
0.538			X		X	X	X
0.518		X	X		X	X	X
0.517			X	X	X	X	X
0.513	X		X	X	X		X
0.513	X		X		X		X

The 10 best correlations between the multivariate pattern of community composition and abundance and environmental variables. For each correlation, the variables whose multivariate pattern resulted in the correlation are denoted by X

**Fig. 2:**
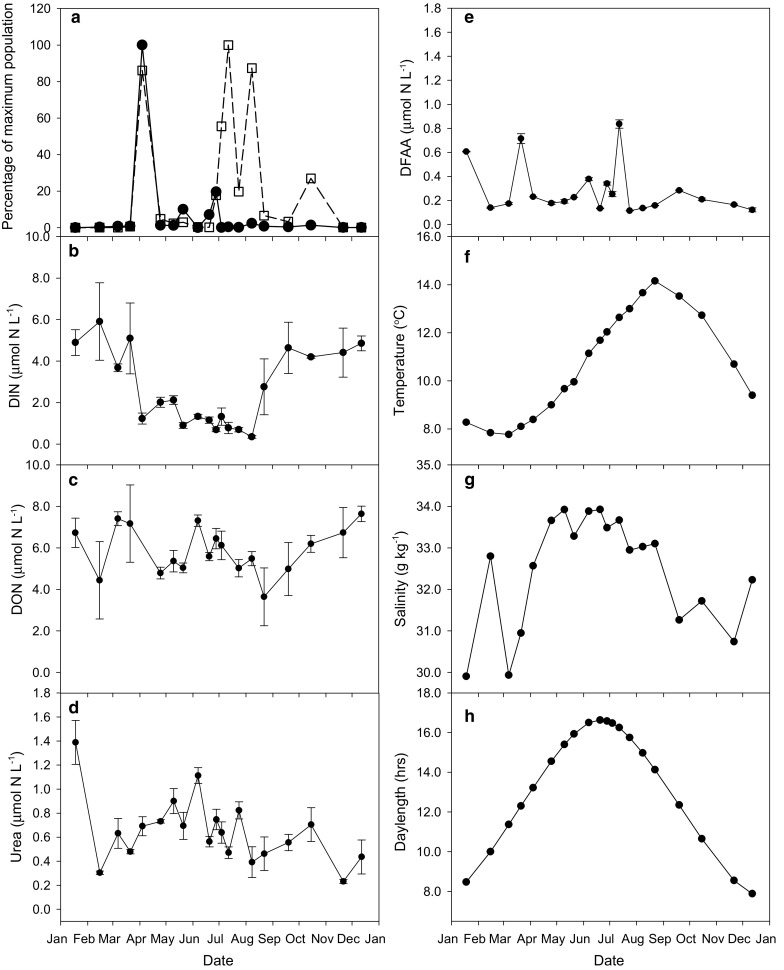
Seasonal patterns in (**a**) percentage of maximum recorded abundance of the spring group (*solid line*, *closed circles*) and summer/autumn group (*dashed line*, *open squares*) and the parameters with which the multivariate pattern of community composition and abundance of 6 selective taxa representative of these two groups were correlated: **b** DIN, **c** DON, **d** urea, **e** DFAA, **f** temperature, **g** salinity, **h** daylength/photoperiod. *Error bars* are standard errors. Please note the change in scale from DIN and DON to urea and DFAA

These seven environmental variables had distinct seasonal patterns (Fig. 2). Concentrations of DIN were highest in winter, decreased in April after the spring bloom, and remained low until the end of August and then increased to winter levels (Fig. 2b). NO_3_^−^ concentrations accounted for most of the seasonal variability in DIN while NH_4_^+^ concentrations remained relatively stable around 0.5 μmol N L^−1^ throughout the year except for a peak in late summer/early autumn. Concentrations of DON were high in winter, autumn and mid-summer, and low in late spring and late summer (Fig. 2c). Urea concentrations reflected the mid-summer highs of DON concentrations, but had only a single high value in winter (Fig. 2d). Concentrations of DFAA were higher in early spring and mid-summer with a single high value in winter at the same time as urea (Fig. 2e). Temperature was lowest in March and highest in late August. Salinity was lowest with relatively high fluctuations in autumn and winter, and highest with relatively small fluctuations in spring and summer. Daylength was lowest on the winter Solstice in December and highest on the summer Solstice in June.

**Fig. 3:**
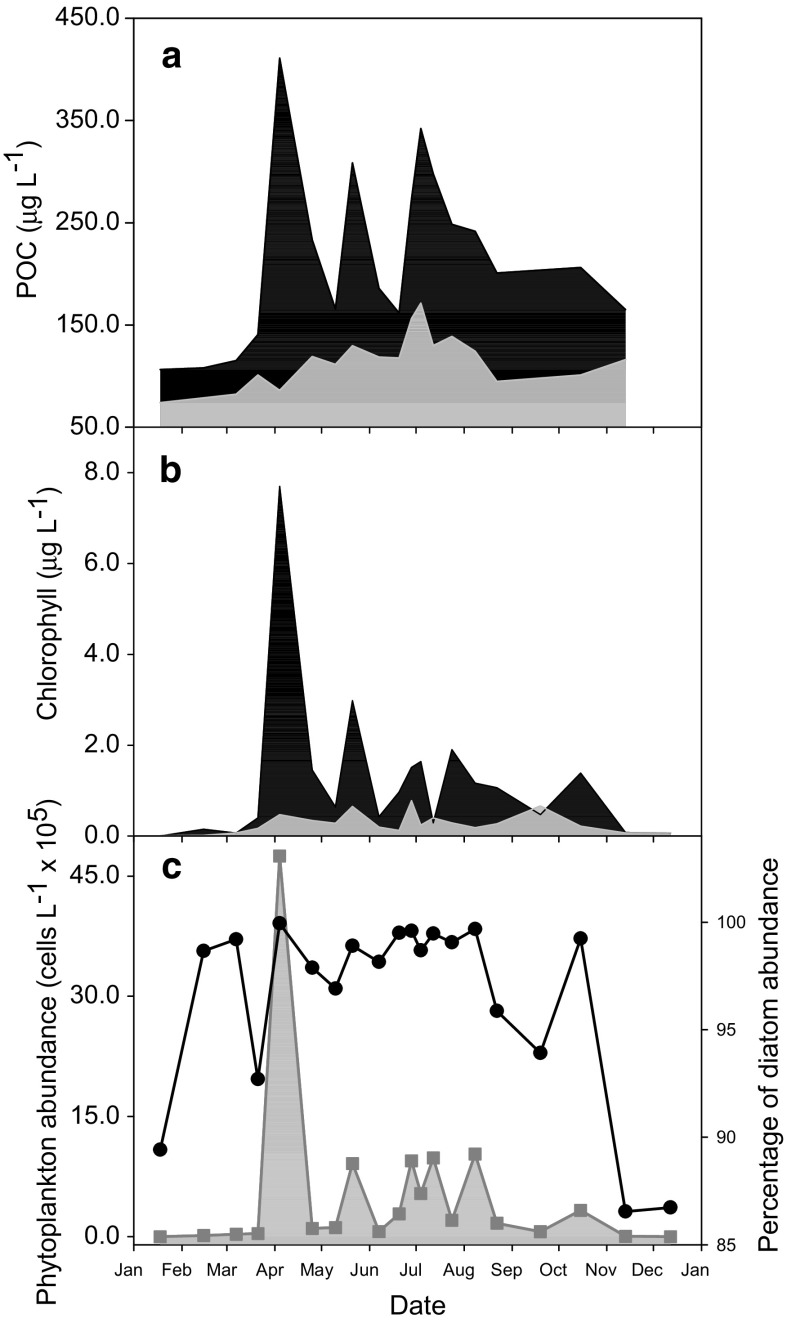
Seasonal microbial and phytoplankton biomass and abundance in Loch Creran in 2012. **a** Microbial biomass of the >10 μm size fraction (*black area*) and the <10 μm size fraction (*grey area*). The two areas together make up the microbial biomass of the <200 μm fraction. **b** Phytoplankton biomass, as chlorophyll concentration, of the >10 μm size fraction (*black area*) and the <10 μm size fraction (*grey area*). The two areas together make up the phytoplankton biomass of the <200 μm fraction. **c** Phytoplankton abundance of all enumerated taxa (*grey area, grey squares*), and diatom abundance as a percentage of the total phytoplankton abundance (*solid line, black circles*)

The taxa enumerated by light microscopy were larger diatoms and dinoflagellates (mostly >10 μm in diameter), and their collective abundance in Loch Creran is referred to hereafter as phytoplankton abundance, POC concentrations as microbial biomass and Chl concentrations as phytoplankton biomass. Morphotype groups of the genera *Pseudo*-*nitzschia* Peragallo (Fehling et al. 2006) and *Chaetoceros* Ehrenberg (Hasle and Syvertsen 1997) were also identified. Microbial biomass, phytoplankton biomass and phytoplankton abundance in Loch Creran in 2012 were highest in spring with smaller blooms evident in summer and late autumn (Fig. 3). POC and Chl concentrations of the <10 μm size fraction remained relatively stable (74–171 µg POC L^−1^ and 0.01–0.8 μg Chl L^−1^) throughout the year compared to the >10 μm fraction (29–325 µg POC L^−1^ and 0–7.3 μg Chl L^−1^). However the former made a substantial contribution (21–73% for POC and 6–100% for Chl) to total microbial biomass (1–200 μm size fraction): higher when biomass was low, but lower during the spring and summer/autumn blooms, for which the >10 μm fraction was mainly responsible (Fig. 3a, b). Diatom abundance as a percentage of total phytoplankton abundance exceeded 85% in all samples (Fig. 3c).

The presence and timing of maximum abundance of different diatom and dinoflagellate taxa varied seasonally (Table 3). The predominant diatoms and dinoflagellates were *Skeletonema* spp. Greville and *Scrippsiella* spp. Balech ex Loeblich III with peaks in their abundance in spring (34.8 × 10^5^ cells L^−1^) and summer (4 × 10^3^ cells L^−1^) respectively. The highest abundances across all enumerated taxa were reached by *Skeletonema* spp. and *Chaetoceros* spp. (44 × 10^4^ cells L^−1^) which encompassed both their coastal and oceanic morphotypes (Hasle and Syvertsen 1997).

**Table 3:** Seasonal distribution of selected diatom and dinoflagellate taxa

	18 Jan	15 Feb	07 Mar	21 Mar	04 Apr	25 Apr	10 May	21 May	07 Jun	20 Jun	28 Jun
Diatoms											
*Pseudo-nitzschia* spp *. group delicatissima*		O	O	O	X	O	O	O		O	O
*Skeletonema* spp.	O	O	O	O	X	O	O	O	O	O	O
*Thalassiosira* spp.	O	O	O	O	X	O	O	O	O	O	O
*Cylindrotheca* spp.	O	O	O	O	X	O	O	O	O	O	O
*Chaeotoceros* spp*. (coastal)*	O	O	O	O	X	O	O	O	O	O	O
*Lauderia* spp.				O	X	O	O	O	O		
*Leptocylindrus minimus*						O	X	O	O	O	O
*Dactyliosolen* spp.							O	X	O	O	O
*Ceratulina* spp.						O	O	X	O	O	O
*Leptocylindrus danicus*							O	O	X		O
*Guinardia delicatula*						O	O	O	X	O	O
*Pseudo-nitzschia* spp *. group seriata*					O		O	O	O	O	O
*Chaeotoceros* spp*. (oceanic).*					O		O	O	O	O	O
*Chaetoceros* spp.	O	O	O	O	O	O	O	O	O	O	O
*Eucampia* spp.								O			O
*Paralia* spp.	O	O	O	O	O	O		O		O	O
Predominant diatom	Par	Skel	Skel	Skel	Skel	Thal	Skel	Skel	Lepd	Skel	Skel
Dinoflagellates											
*Prorocentrum minimum*							O	X	O	O	O
*Protoperidinium* spp.		O	O	O	O	O	O	X	O	O	O
*Katodinium* spp.				O	O	O	O	O	O	O	X
*Scrippsiella* spp.		O	O	O	O	O	O	O	O	O	O
*Gyrodinium* spp.	O	O	O	O	O	O	O	O	O	O	O
*Ceratium* spp.											
*Gonyaulax* spp.			O	O	O	O	O	O			
*Heterocapsa* spp.			O	O	O	O	O	O	O	O	O
*Gymnodinium* spp.	O	O	O		O	O	O	O	O	O	
Predominant dinoflagellate	Gym	Gyr	Scrip	Scrip	Scrip	Scrip	Kat	Pro	Kat	Kat	Scrip

	04 Jul	12 Jul	24 Jul	08 Aug	22 Aug	19 Sep	15 Oct	13 Nov	12 Dec	Maximum (cells L^−1^)
Diatoms										
*Pseudo-nitzschia* spp *. group delicatissima*	O	O	O	O	O	O	O	O	O	123,897
*Skeletonema* spp.	O	O	O	O	O	O	O	O	O	3,480,378
*Thalassiosira* spp.	O	O	O	O	O	O	O	O	O	246,386
*Cylindrotheca* spp.	O	O	O	O	O	O	O	O	O	8280
*Chaeotoceros* spp*. (coastal)*	O	O	O	O	O	O	O	O	O	437,538
*Lauderia* spp.				O	O	O	O			13,840
*Leptocylindrus minimus*	O	O	O	O	O	O	O			27,760
*Dactyliosolen* spp.		O	O	O	O	O	O			326,637
*Ceratulina* spp.		O	O	O	O	O	O	O		141,334
*Leptocylindrus danicus*	O	O	O	O	O	O				25,800
*Guinardia delicatula*	O	O	O	O	O	O	O			9440
*Pseudo-nitzschia* spp *. group seriata*	O	X	O	O	O	O	O	O		64,981
*Chaeotoceros* spp*. (oceanic).*	O	X	O	O	O	O	O	O		99,096
*Chaetoceros* spp.	O	X	O	O	O	O	O	O	O	441,870
*Eucampia* spp.	O	O	O	O	X	O	O			4420
*Paralia* spp.	O	O	O			X	O	O		760
Predominant diatom	Chae	Chae	Chae	Chae	Chae	Chae	Chae	Skel	Skel	
Dinoflagellates										
*Prorocentrum minimum*	O	O	O	O	O	O	O	O		5320
*Protoperidinium* spp.	O	O	O	O	O	O	O	O		2540
*Katodinium* spp.	O	O	O	O	O	O	O	O		1140
*Scrippsiella* spp.	X	X	O	O	O	O	O	O		4000
*Gyrodinium* spp.	O	O	O	X	O	O	O	O	O	620
*Ceratium* spp.		O	O	O	X	O	O			420
*Gonyaulax* spp.	O	O			X	O	O	O		220
*Heterocapsa* spp.	O	O	O	O	O	X	O		O	1180
*Gymnodinium* spp.	O	O	O	O	O	X	O	O	O	480
Predominant dinoflagellate	Scrip	Scrip	Scrip	Scrip	Scrip	Het	Scrip	Gym	Gym	

Open circles show presence and crosses show peaks in abundance. Also shown are maximum recorded abundance for each taxon and predominant diatom and dinoflagellate taxa in each sample. Underlined taxa belong to the subset of 15 most important taxa. Par: *Paralia* spp.; Skel: *Skeletonema* spp.; Thal: *Thalassiosira* spp.; Lepd: *Leptocylindrus danicus*; Chae: *Chaetoceros* spp.; Gym: *Gymnodinium* spp.; Gyr: *Gyrodinium* spp.; Scrip: *Scrippsiella* spp.; Kat: *Katodimium* spp.; Pro: *Prorocentrum minimum*; Het: *Heterocapsa* spp.

**Fig. 4:**
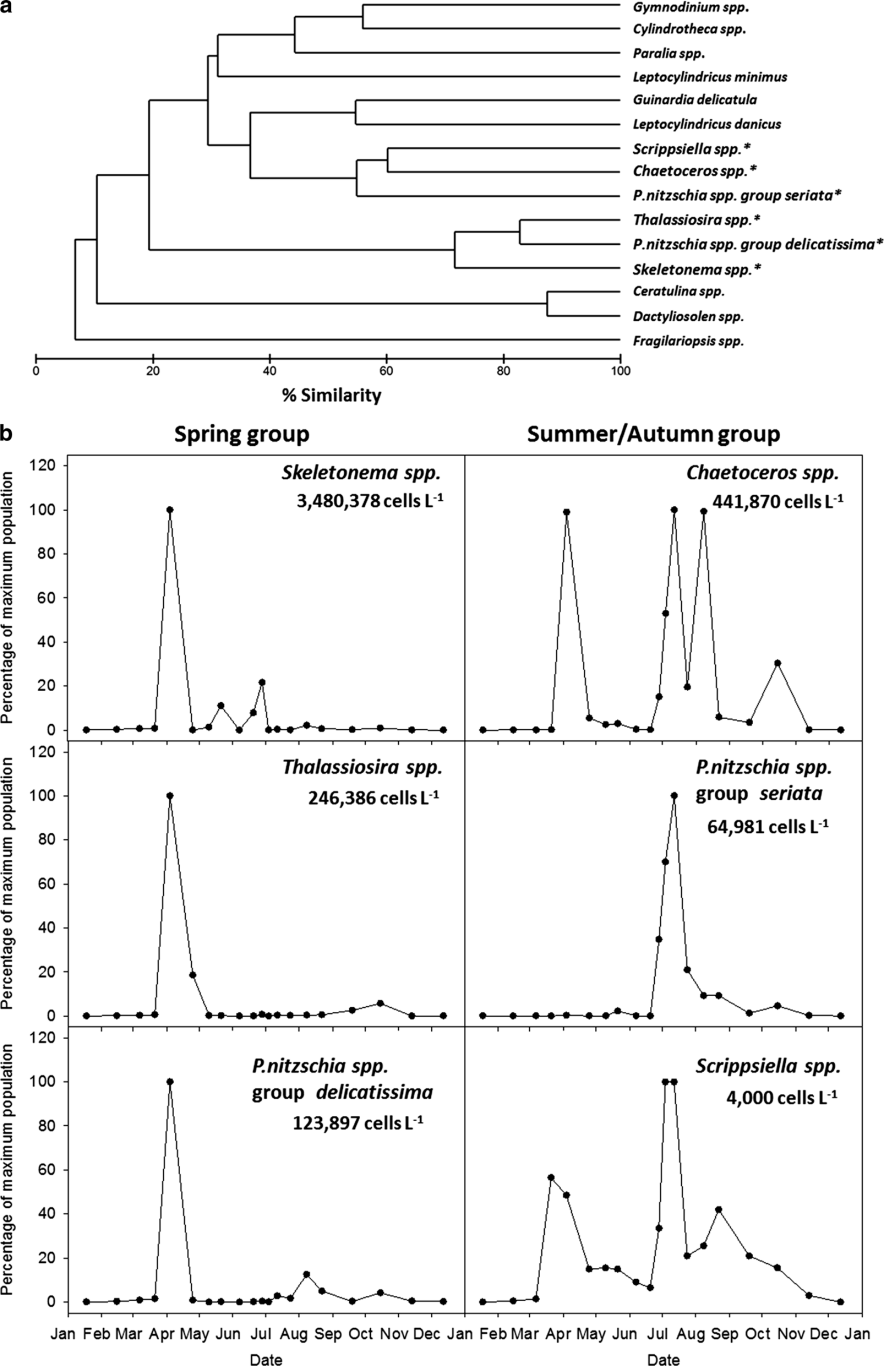
Group classification of the 15 most important taxa in Loch Creran in 2012. **a** Dendrogram generated by cluster analysis based on a Bray–Curtis similarity matrix of abundance data from the subset of 15 taxa. **b** Seasonal patterns in the percentage of maximum recorded population for 6 selected taxa (*asterisks*) representative of groups identified from the cluster analysis. Maximum recorded abundance for each taxon is shown (cells L^−1^)

The dendogram generated from Cluster analysis (Fig. 4a) showed that the subset of 15 most important taxa could be divided into several groups based on the similarity in their seasonal distribution. At <10% similarity between groups (i.e. >90% dissimilarity between groups) *Fragilariopsis* spp. Hustedt stood out in its own group and at ~10% *Ceratulina* spp. Peragallo ex Schütt and *Dactyliosolen* spp. Castracane also formed a separate group. At a cut-off point of ~20% similarity between groups (i.e. ~80% dissimilarity), the remaining 12 phytoplankton taxa could be split into two more groups: a group containing the taxa *Skeletonema* spp., *Thalassiosira* spp. Cleve, and *Pseudo*-*nitzschia* spp. group *delicatissima*, and a group containing the remaining 9 taxa.

The latter group could be split into three further groups at 40% similarity between them (Fig. 4a), one of which contained the taxa *Chaetoceros* spp., *Scrippsiella* spp., and *Pseudo*-*nitzschia* spp. group *seriata.* These 6 taxa were selected for subsequent analysis based on their overall predominance among all enumerated taxa (Table 3) and strong separation in the Cluster (Fig. 4a) and MDS analysis, and were representative of two groups (Table 3; Fig. 4): a spring group containing *Skeletonema* spp., *Thalassiosira* spp., and *Pseudo*-*nitzschia* spp. group *delicatissima* and a summer/autumn group containing *Chaetoceros* spp., *Scrippsiella* spp., and *Pseudo*-*nitzschia* spp. group *seriata*. Less abundant diatoms and dinoflagellates of note were *Leptocylindus danicus* Cleve, which peaked in early summer, and *Prorocentrum minimum* Pavillard which, despite reaching higher maximum abundance than *Scrippsiella* spp., only predominated briefly in early spring (Table 3).

The seasonal patterns in abundance of these groups differed (Fig. 4b). Taxa from the first group had peaks and increases in abundance in spring, while taxa from the second group, despite some also having high abundances in spring, had peaks and increases in abundance mostly in late summer and early autumn. The most abundant taxa in the spring group and summer/autumn groups were *Skeletonema* spp. and *Chaetoceros* spp. respectively (Fig. 4b).

**Fig. 5:**
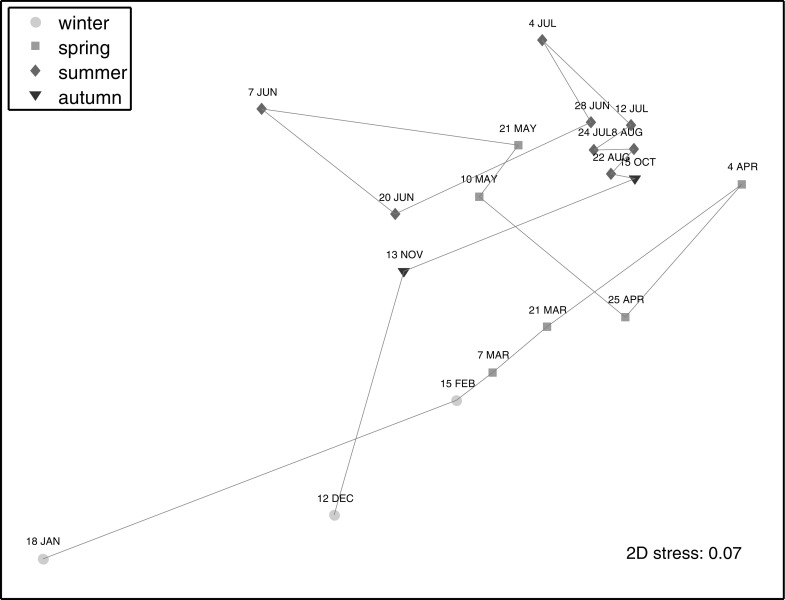
MDS plot of log(x + 1) transformed abundance of the 6 selected taxa representative of groups identified by cluster analysis based on a Bray–Curtis similarity matrix. *Lines* connect consecutive sample dates

Changes in community composition and abundance of these 6 taxa were depicted on an MDS plot (Fig. 5). Statistical comparisons between the multivariate pattern in the community composition of the 6 selected taxa and that of all enumerated taxa revealed that they were very similar (p = 0.001), suggesting that the MSD plot featuring the 6 selected taxa was representative of the entire community. Distances between samples represent their dissimilarity, with larger distances denoting larger dissimilarity. Winter samples transitioned from the left bottom corner of the plot to spring samples on the right side of the plot. Spring samples were not obviously clustered together; most likely because the duration of the spring bloom was brief (Figs. 3, 5). In contrast, late summer and autumn samples were closely clustered together in the top right corner of the plot (Fig. 5). In turn, by November the population had transitioned back to its winter position.

**Fig. 6:**
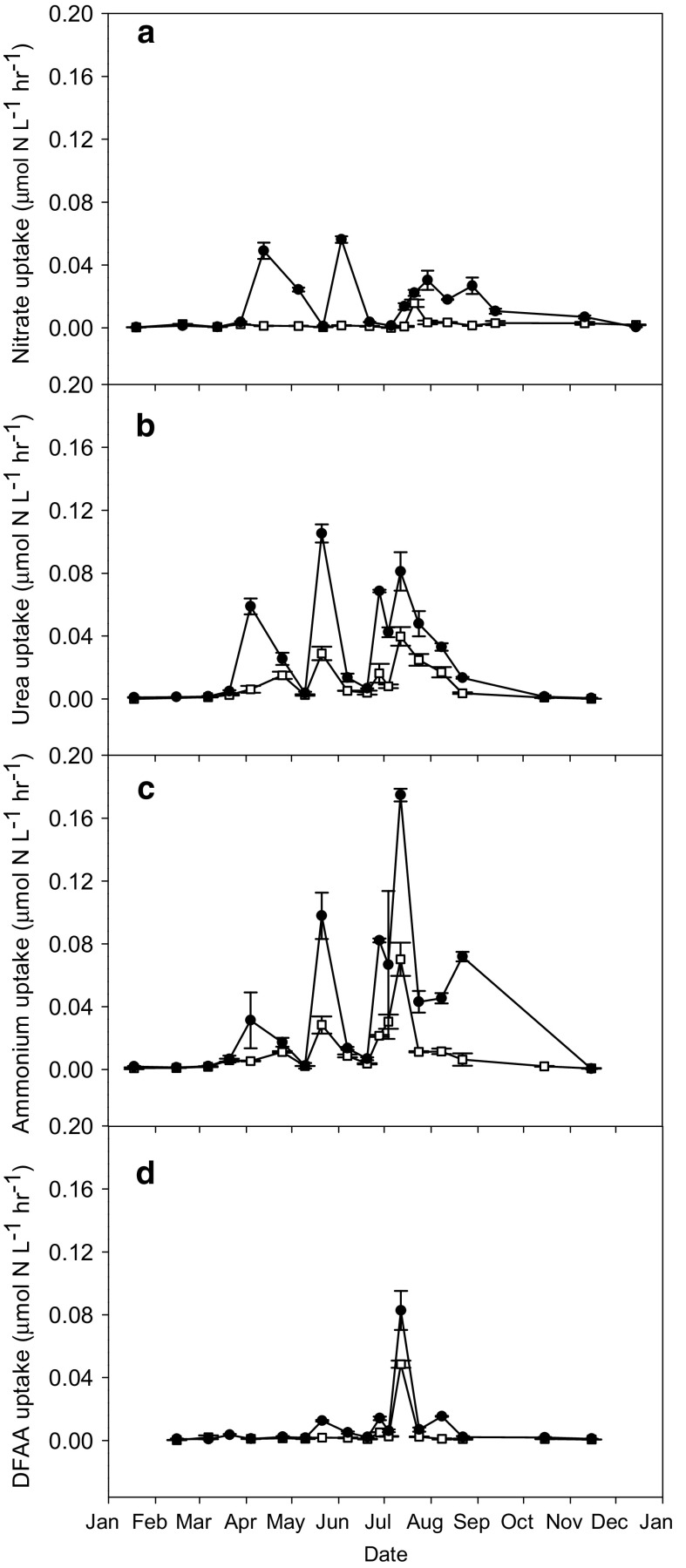
Seasonal patterns in N uptake by the <200 μm size fraction (*closed circles*) and the <10 μm size fraction (open squares) from NO_3_^−^, urea, NH_4_^+^, and DFAA in Loch Creran in 2012. *Error bars* are standard errors

**Fig. 7:**
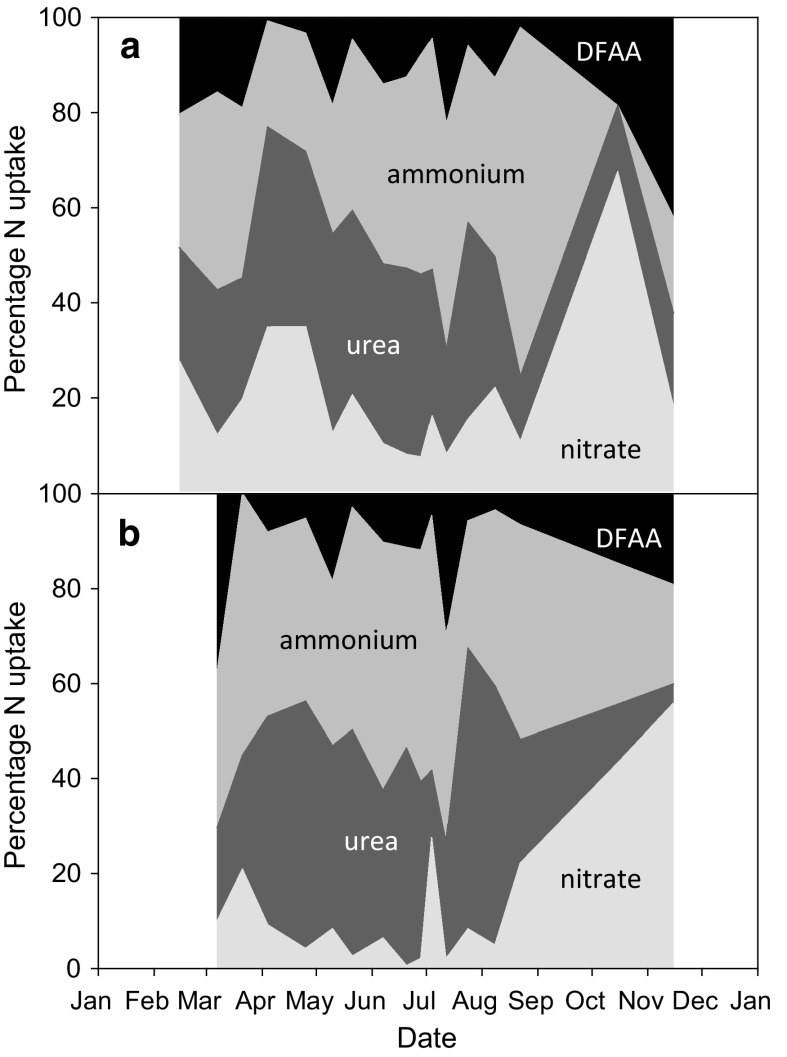
Seasonal patterns in the proportion of total measured N uptake accounted for by NO_3_^−^, urea, NH_4_^+^, and DFAA in **a** the <200 μm size fraction and **b** the <10 μm size fraction in Loch Creran in 2012. **a** NO_3_^−^ (8–68%), urea (14–42%), NH_4_^+^ (0–73%), DFAA (1–42%). **b** NO_3_^−^ (1–56%), urea (4–59%), NH_4_^+^ (21–55%), DFAA (0–38%)

Seasonal peaks in N uptake rates (Fig. 6) coincided with peaks in microbial biomass and phytoplankton biomass and abundance, but the absolute magnitude and relative uptake varied seasonally and between different N compounds (Figs. 6, 7). NO_3_^−^ uptake rates by the microbial community were highest during the spring bloom and in late spring and lower in summer (Fig. 6a). Urea uptake rates reached peaks during the spring bloom, late spring, and summer (Fig. 6b). NH_4_^+^ and DFAA uptake rates were highest in summer (Fig. 6c, d). Generally, the highest N uptake rates were those of NH_4_^+^, followed by urea, DFAA and NO_3_^−^. During the spring bloom, urea and NO_3_^−^ uptake rates were highest followed by those of NH_4_^+^ and DFAA while in late spring urea uptake rates were also highest followed by those of NH_4_^+^, NO_3_^−^ and DFAA (Figs. 6, 7a). In summer, NH_4_^+^ uptake rates were highest, followed by those of urea. The DFAA summer uptake rates showed substantial increases for the first time, whereas NO_3_^−^ uptake rates were generally lower than earlier in the season.

The contribution of the <10 μm size fraction to total microbial N uptake (Fig. 6) was >50% at low uptake rates and <50% during periods of high uptake rates in spring and summer/autumn, although this varied between different N compounds. Specifically, the contribution (median %) of the smaller size fraction to compound specific N uptake was lowest for NO_3_^−^ (29%) and highest for the reduced N forms NH_4_^+^ (46%), urea (44%) and DFAA (44%), especially in summer (Fig. 6). Generally, the smaller microbes took up relatively more urea (up to 59%) and NH_4_^+^ (up to 55%) during the productive season whereas NO_3_^−^ (up to 56%) became more important during autumn and winter (Fig. 7b) when its concentrations were high.

## Discussion

### Relationships with environmental parameters

Sequence, or the rate of introduction of allochthonous populations (Smayda 1980), may have had an effect in the spring versus summer/autumn grouping of taxa in Loch Creran in 2012. *Chaetoceros* spp. was present as two different morphotypes, one that is mainly coastal and one that is mainly oceanic (Hasle and Syvertsen 1997). The abundance of the coastal morphotype peaked in spring and summer and was about four times higher than that of the oceanic morphotype which only peaked in summer (Table 3; Fig. 4b). It is possible that the oceanic morphotype was transported to Loch Creran from offshore waters, as waters of the Firth of Lorne exchange with more ocean influenced shelf water (Fehling et al. 2012, 2006 and references therein). However, once in Loch Creran, its growth would be subjected to the same local environmental conditions that affect the growth of the indigenous flora.

Phytoplankton succession can be driven by several physical and chemical parameters. Those that correlated with the multivariate pattern in community composition and abundance in Loch Creran were, in order of statistical importance: urea, daylength, temperature, DFAA, salinity, DON, and DIN (Table 2). Temperature and daylength varied seasonally in a typical and predictable sinusoidal fashion (Fig. 2), and are frequently identified as important factors influencing phytoplankton species composition (Boyd et al. 2013; Eppley 1972; Fehling et al. 2005; Karentz and Smayda 1984; Lomas and Glibert 1999a, b).

Salinity in Loch Creran varies with the intensity and frequency of rainfall (unpublished data). Nutrient concentrations in temperate coastal areas that receive freshwater inputs may be expected to vary with salinity, with the relationship being stronger in winter and weaker in spring and summer when biological growth exerts a greater influence. In Loch Creran, Solórzano and Ehrlich (1979) found inverse correlations between salinity and the concentrations of DIN, DIS and DON, especially during the autumn and winter months. The effect of salinity in these months on nutrient concentrations is also evident in this study: from the end of August salinity decreased over the autumn and DIN (Fig. 2b), DON (Fig. 2c), DIP and DIS (not shown) concentrations began to increase back to their winter levels. Also, in winter there was an obvious relationship between salinity and DON concentrations, with an increase in salinity on 15 February coinciding with a decrease in DON concentrations.

In several temperate coastal and shelf sea systems, such as the North Sea (Johnson et al. 2012; Van Engeland et al. 2010), English Channel (Butler et al. 1979), Irish Sea (Moschonas et al. 2015) and Chesapeake Bay (Bronk et al. 1998), total DON concentrations follow a seasonal trend in which DON increases in spring or summer, during or following the drawdown of DIN by phytoplankton production, and is subsequently decreases over the course of several weeks or months.

During this study, DON concentrations reached a maximum at the beginning of June and then gradually decreased to a minimum at the end of August, like the gradual DON drawdown observed in the above studies. The bulk of DON that was gradually remineralised during the summer months can be classified as semi-labile DON. Generally, semi-labile DON has turnover times of weeks to months (Carlson and Hansell 2015; Johnson et al. 2012; Lønborg et al. 2009a, b) and is accessible mainly to bacteria (Bronk et al. 2007).

Urea and DFAA concentrations remained low throughout the year and accounted for between 6 and 30% of total DON concentration. Urea and DFAA are labile DON compounds with high turnover rates which prevent them from accumulating in high concentrations (Bronk et al. 2007; Sipler and Bronk 2015). Semi-labile and refractory DON compounds such as proteins, amino polysaccharides and humic substances turn over much slower and therefore have higher concentrations, although there are now indications that some humic substances may be highly bioavailable (See et al. 2006). The turnover times for urea and DFAA between March and October were 1–34 and 1–19 h respectively, with both compounds having turnover times of less than 7 h on most dates. This means that during the productive season the entire urea and DFAA stocks in Loch Creran were turned over in less than a day and a half, and most often in less than 7 h.

### Seasonal phytoplankton distribution

Within the constraints of inter-annual variability, phytoplankton biomass and abundance in Loch Creran exhibits a seasonal cycle characteristic of temperate coastal waters, with a bloom in early spring and smaller blooms in summer and early autumn (Tett and Wallis 1978). In 2012, the microbial and phytoplankton biomass of the <10 μm size fraction remained relatively constant, while the biomass of the >10 μm size fraction fluctuated in time, and was mostly responsible for the blooms in spring and summer/autumn (Fig. 3a, b). The biomass of the smaller phytoplankton size fractions is relatively stable over time (Chisholm 1992; Raimbault et al. 1988) because it is generally more tightly controlled by grazing (Barber and Hiscock 2006; Fogg 1991; Glibert 1993; Smetacek 2002). In contrast, the biomass of the larger size fractions oscillates considerably over time (Fenchel 1988; Fogg 1991; Smetacek 2002) affected by environmental parameters such as NO_3_^−^ concentration (Maguer et al. 2009; Malone 1971; Wilkerson et al. 2000).

The abundance of phytoplankton taxa that were identified and enumerated by light microscopy were predominantly larger cells >10 μm and were comprised mainly of diatoms. Diatoms are thought to prefer well-mixed, nutrient replete conditions (although see Kemp and Villareal (2013) for a different view), in contrast to stratified nutrient-limited conditions that are thought to favour dinoflagellates (Falkowski et al. 2004; Margalef 1978; Tozzi et al. 2004). There are numerous physiological reasons why diatoms may thrive under NO_3_^−^-rich conditions (Glibert et al. 2016 and references therein). For example, they generally have more NO_3_^−^ transporters than other phytoplankton taxa and higher rates of NO_3_^−^ reductase activity (e.g., Song and Ward 2007; Lomas and Glibert 1999b). Diatoms also dominate under conditions of cool temperature. While cool temperatures may be less favourable for photosynthesis, diatoms are thought to be able to regulate overall cellular energy balance in cool water through the reduction of NO_3_^−^ to NO_2_^−^ and even NH_4_^+^ via a non-assimilatory pathway that complements their use of NO_3_^−^ in a nutritionally assimilatory mode (e.g., Lomas and Glibert 1999a, b; Parker and Armbrust 2005; Kamp et al. 2011; Glibert et al. 2016).

Loch Creran is a shallow and tidally mixed fjord typical of Scottish waters, which only stratifies periodically because of lower salinity at the surface (Fehling et al. 2006; Gowen et al. 1983), and has nutrient concentrations that rarely fall below their limit of detection, even in the relatively dry summer months (Fig. 2). The intermittent mixing and stratification and perhaps resupply of new N may be why diatoms predominate in the cool, NO_3_^−^-rich waters of the loch. However, the phytoplankton community in coastal waters is generally subject to long-term variability (e.g. Bresnan et al. 2009), and the balance between diatoms and flagellates in Loch Creran may be changing (Whyte et al. 2016).

The 6 most abundant taxa, *Skeletonema* spp., *Thalassiosira* spp., *Pseudo*-*nitzschia* spp. group *delicatissima, Chaetoceros* spp., *Scrippsiella* spp., and *Pseudo*-*nitzschia* spp. group *seriata*. are prominent members of the phytoplankton community in Scottish and other temperate coastal waters worldwide; and similar spring-summer/autumn successional patterns involving them have been documented before (Borkman and Smayda 2009; Bresnan et al. 2009; Gowen, et al. 2012b; Henriksen 2009; Smayda 1980). The non-toxic *Pseudo*-*nitzschia* spp. group *delicatissima* occurred mainly in spring, and the potentially toxic *Pseudo*-*nitzschia* spp. group *seriata* mainly in summer (Table 2; Fig. 4b). It is of note that *P. minimum* is also an important HAB species (Heil et al. 2005; Glibert et al. 2012), although its occurrence was comparatively short-lived. Some *Pseudo*-*nitzschia* species can produce the neurotoxin domoic acid which, when concentrated by filter-feeding shellfish and is ingested by humans, can result in Amnesic Shellfish Poisoning (Davidson et al. 2011). The Scottish west coast is a region of shellfish aquaculture and *Pseudo*-*nitzschia* are actively monitored. Our findings agree with the pattern of *Pseudo*-*nitzschia* dominance observed by Fehling, et al. (2006) in the Firth of Lorne, adjacent to Loch Creran.

### Seasonal patterns in uptake of different forms of N

In temperate coastal systems, the magnitude of N uptake, the main form of N utilised, and the predominant phytoplankton taxa can vary seasonally. For example, in Chesapeake Bay, evidence shows that phytoplankton take up mainly allochthonous new N, as NO_3_^−^, in spring and autochthonous regenerated N in summer and autumn (McCarthy et al. 1977; Bronk et al. 1998). In the Norwegian Oslofjord, Paasche and Kristiansen (1982) found a similar trend and observed that N uptake rates were highest in the summer. Berg et al. (2003) investigated relationships between phytoplankton functional groups and seasonal uptake of different N forms in the brackish Gulf of Riga, and found that NO_3_^−^ uptake was correlated with the relative abundance of diatoms, while regenerated N in the form of NH_4_^+^, urea and amino acids was correlated with that of dinoflagellates, cryptophytes, and cyanobacteria. Similar reports of dichotomous use of different forms of N have been frequently reported. Glibert et al. (2004) reported the same phenomenon from Florida Bay, as did Domingues et al. (2011) for a tidal fresh estuary. Similar observations have been reported from mesocosm experiments variably enriched with different forms of N (e.g. Donald et al. 2011, 2013; Fawcett and Ward 2011; Glibert et al. 2014b, 2016).

Our results demonstrated that urea and NO_3_^−^ were used at highest rates in spring, NH_4_^+^ was used at highest rates in summer/autumn, and overall N uptake rates were generally highest in summer. The generally accepted view is that new N as NO_3_^−^ is important in spring and regenerated N in summer (e.g., Glibert 1997 and references therein). Urea uptake rates may be expected to be relatively low during the spring bloom given the literature status of urea as a regenerated nutrient. However, this study shows that urea uptake rates can be highest relative to other N sources during the spring bloom, up to 44% of the total N uptake. There are indications that this may also be the case during spring in the Canadian Arctic (Simpson et al. 2013) and during an induced *Phaeocystis* bloom in a Norwegian fjord (Sanderson et al. 2008). The recent identification of a urea cycle in diatoms may be relevant in this regard (e.g., Armbrust et al. 2004; Allen et al. 2011); whereas the urea cycle in mammals serves to remove excess N, in diatoms it appears to also play an important role in the fixation of C and N and the rapid metabolic response following short-term nutrient withdrawal or introduction.

In addition to the high absolute and relative urea uptake rates during the spring bloom (Figs. 6b, 7a), a considerable portion of the total DON pool contributed potentially new N from winter DON highs to spring production as seen from the drop in DON concentration (Fig. 2c). We estimate that the drawdown of DON during the spring bloom could have contributed up to 2.6 ± 0.4 μmol N L^−1^ compared to 4.5 ± 1.9 μmol N L^−1^ from NO_3_^−^, i.e. 37 and 63% respectively of the total measured dissolved N drawdown.

Urea uptake remained relatively high in summer and autumn (up to 41% of the total measured dissolved N drawdown, Fig. 7a), and this finding adds to evidence suggesting that urea is of importance for phytoplankton N nutrition year-round (Glibert et al. 2006, 2014a; Lomas et al. 2002; Solomon et al. 2010). In addition, our results uniquely suggest that this is the case even in a near-pristine location leading to the conclusion that urea inputs from anthropogenic sources may boost processes which are naturally occurring and highly important to local production. It is of note that rates of NO_3_^−^ uptake herein may potentially have been underestimated due to the necessity of conducting the incubations in the afternoon and the known diel component of NO_3_^−^ uptake (e.g., Berges et al. 1995; Glibert et al. 1991, 2016).

The <10 μm size fraction contributed substantially to total N uptake (Fig. 6), and was higher for regenerated N rather than NO_3_^−^ (Fig. 7). Evidence suggests that although primary production by the smaller size fractions is important and often dominant, especially in oligotrophic regions, the contribution of the larger size fraction increases and often dominates in coastal waters, where NO_3_^−^ levels are higher (Lomas and Glibert 1999a; Maguer et al. 2009; Malone 1971; Wilkerson et al. 2000). Also, the smaller size fractions appear to contribute more to regenerated N uptake, while the larger size fractions to new N uptake (Glibert et al. 2016; Joint et al. 1986; Joubert et al. 2011). Our data show that in the <10 μm fraction, NO_3_^−^ contributed little to N uptake (up to 28% during spring and summer but generally much lower, Fig. 7b), but NH_4_^+^ (up to 55%), urea (up to 59%), and DFAA (up to 38%) did considerably during spring and summer (Fig. 7b), especially in the summer when regenerated N uptake rates were highest (Fig. 6). There are several possible physical (e.g. diffusion limitation) and biochemical (e.g. assimilation pathways) reasons why different size classes may prefer different forms of N, and this is an area of research that requires further study.

Bacteria are important components of the microbial community and probably contributed to N uptake and interacted with phytoplankton. The traditional model of N uptake in the marine environment used to classify inorganic N uptake as a phytoplankton process and organic N uptake as a bacterial process, but the paradigm has now changed (Zehr and Ward 2002). Phytoplankton can clearly assimilate organic N and bacteria inorganic N. Specifically, bacteria tend to take up more reduced than oxidised inorganic N while at the same time regenerating NH_4_^+^; also, different bacterial phylogenetic groups may be responsible for uptake of different N compounds (Kirchman 2000). Additionally, bacterially produced extracellular enzymes can break down complex N compounds which in turn can be taken up by both bacteria and adjacent phytoplankton (Arnosti 2011).

The differences in N uptake between spring and summer/autumn were associated with two statistically separate phytoplankton groups: the spring group was associated with increased uptake rates of new N and the summer/autumn group with increased uptake rates of regenerated N. Regenerated inorganic N and DON have been connected to the development of HABs. Supporting evidence comes from laboratory and field studies which found that regenerated N is associated with dinoflagellates (most HAB species are dinoflagellates), and that DON supports the growth of some HAB species (Davidson et al. 2012 and references therein). In this study, uptake rates of regenerated inorganic N were highest in summer/autumn when the potentially toxic diatom *Pseudo*-*nitzschia* spp. group *seriata* was most abundant.

## Summary and conclusion

N uptake rates and total DON concentration patterns suggested high utilisation throughout the year with a mainly labile profile in spring and semi-labile profile in summer. Urea uptake rates were high in both spring and summer/autumn, even when water column concentrations were low while DFAA uptake rates were generally very low but increased briefly mid-summer. The higher urea uptake rates and drawdown of total DON during the spring bloom were unexpected and suggest that this fraction of DON not only naturally contributes to regenerated production but can also contribute substantially to new production. The >10 μm fraction contributed most to the uptake of new N rather than regenerated N, especially in summer/autumn. In contrast the <10 μm fraction took up mostly regenerated N. The <10 μm fraction formed a constant background of microbial and phytoplankton biomass, important at low total biomass levels, but exceeded by the larger size fraction during phytoplankton blooms in spring and summer/autumn. We identified two statistically significant phytoplankton groups, mostly belonging to the larger size fraction, with peaks in spring vs summer/autumn. Their seasonal patterns of community composition and abundance were significantly correlated with urea, daylength, temperature, DFAA, salinity, DON, and DIN. This comprehensive study of seasonal DON biogeochemistry clearly showed that DON was important in the yearly N cycle, for phytoplankton N nutrition, and as an environmental variable that influences phytoplankton seasonal distribution and abundance in a near-pristine coastal location. This supplements similar findings from regions with medium (e.g. Irish Sea) and high (e.g. Chesapeake Bay) N inputs and highlights that DON should be widely studied not only in the context of anthropogenic pressures but also as part of natural ecosystem functioning.
